# Cannabidiol usage, efficacy, and side effects: analyzing the impact of health conditions, medications, and cannabis use in a cross-sectional online pilot study

**DOI:** 10.3389/fpsyt.2024.1356009

**Published:** 2024-02-29

**Authors:** Alicja Anna Binkowska, Natalia Jakubowska, Anna Redeł, Sandra Laskowska, Stanisław Szlufik, Aneta Brzezicka

**Affiliations:** ^1^ Institute of Psychology, Humanitas University, Sosnowiec, Poland; ^2^ Department of Psychology, SWPS University of Social Sciences and Humanities, Warsaw, Poland; ^3^ Nencki Institute of Experimental Biology of the Polish Academy of Sciences, Warsaw, Poland; ^4^ DrugsTeam, NeuroCognitive Research Center, SWPS University of Social Sciences and Humanities, Warsaw, Poland; ^5^ Department of Neurology, Faculty of Health Science, Medical University of Warsaw, Warsaw, Poland

**Keywords:** cannabis, cannabidiol, CBD, anxiety, depression, sleep, health, stress

## Abstract

**Background:**

Products containing cannabidiol (CBD) are attracting attention because of their potential therapeutic benefits and positive impacts on well-being and mental health. Although additional research is needed to understand their effectiveness in treating mental disorders, cross-sectional studies may help identify the factors influencing CBD use patterns. This study examined the impact of variables such as health status, medication use, medical supervision, gender, age, and cannabis use on CBD consumption patterns.

**Materials and methods:**

A self-selected sample (n =267) of current or former CBD users was recruited via social media and participated in an online survey designed to collect data on basic demographics, health status, cannabis use, and CBD usage patterns.

**Results:**

The sample (n = 267) consisted of 68.5% women with an average age of 30.21 years, of which 25.8% reported diagnosed psychiatric disorders and 49.4% reported cannabis use. The top five reasons for using CBD were self-reported stress (65.3%), sleep problems (51.7%), overall improvement in well-being (52.5%), improved mood (44.9%), and anxiety relief (40.9%). Our findings suggest that individuals with psychiatric disorders and those taking psychotropic medications are more likely to use CBD to relieve stress and anxiety. Overall, nearly 70% of the individuals found CBD products to be effective. Sublingual administration was more popular among non-cannabis users, while cannabis users preferred smoking and vaping to CBD administration.

**Conclusion:**

Our results indicate that individuals using CBD for health and wellness reasons believe that it has potential health benefits. Further research using rigorous longitudinal designs is needed to delve deeper into the effectiveness of low-dose CBD and to better understand the therapeutic potential of CBD.

## 1 Introduction

Cannabidiol (CBD), a phytocannabinoid found in *Cannabis sativa*, is gaining popularity. Often described as a non-psychoactive compound, CBD crosses the brain-blood barrier and influences mental processes, such as cognition, mood, and emotions (1–3). Notably, it lacks intoxicating effects akin to delta-9-tetrahydrocannabinol (THC), commonly known as the “high” (4, 5). Cannabis contains numerous active compounds including over 100 cannabinoids and terpenes (6).

Research suggests that CBD has the potential to treat various mental health problems and enhance overall well-being. CBD has attracted interest as a fast-acting antidepressant in preclinical studies (7–9) and has demonstrated anxiolytic effects in clinical studies on patients with social anxiety disorders and healthy adults, with a favorable safety and tolerability profile (1, 2, 10). Simultaneously, CBD has gained popularity as a widely used food supplement, contributing to the growing global cannabidiol market, valued at USD 5.18 billion by 2021 (11). Observational studies have highlighted common reasons for CBD use, including stress relief, improved sleep, and enhanced general health and well-being (12, 13). Patients also use CBD for various medical conditions, such as pain, anxiety, and depression (14, 15), with respondents consistently reporting CBD’s effectiveness in alleviating symptoms. However, the current lack of high-quality evidence precludes recommendations for CBD use for psychiatric disorders (16). Well-designed, longitudinal, and adequately powered preclinical and clinical studies are essential to comprehensively understand CBD’s effectiveness and the treatment protocols for specific psychiatric disorders.

CBD products are usually administered in oils, but they are also available in the form of edibles – baked into brownies and cakes or added to gummies. There are three main types of CBD concentrates available on the market, differing in the cannabinoids they contain. Full-spectrum CBD products contain all of the compounds found naturally in cannabis, including THC – although to adhere to FDA regulation, they can’t contain more than 0.3% of THC. Broad-spectrum CBD products also contain all of the natural cannabinoids however, most of the THC is filtered out, leaving only trace amounts in the finished product. Only CBD isolates, known as ‘pure’ CBD products, contain no THC (17). Currently, most CBD products are considered dietary supplements, with very little regulation over the market (18). Studies estimate that even more than half of commercially available CBD products may contain different doses from what’s reported on the labels (19, 20), which poses a potential risk to people using CBD to self-medicate.

In the U.S., the one form of CBD medication is an FDA-approved prescription drug for seizures. There are also some reports of CBD being potentially useful in the treatment of other symptoms, with currently ongoing, clinical trials testing CBD efficacy in relieving e.g. chronic pain, anxiety, and insomnia (https://clinicaltrials.gov/study/NCT04729244), bipolar depression disorder (https://clinicaltrials.gov/study/NCT05867849), obsessive-compulsive disorder (https://clinicaltrials.gov/study/NCT04978428 autism spectrum disorder (https://clinicaltrials.gov/study/NCT05015439), endometriosis (https://clinicaltrials.gov/study/NCT04527003), or COVID-19 symptoms (https://clinicaltrials.gov/study/NCT04686539).

Ongoing registered clinical trials are examining the effects of CBD across numerous psychiatric and physical conditions, bridging the gap between observed user behavior and evidence-based scientific findings. Concurrently, cross-sectional studies provide valuable insights into CBD usage patterns and identify critical factors for consideration in further research, such as randomized controlled trials (RCTs), as well as in consumer or patient information regarding the potential benefits and risks associated with CBD product usage.

Our study aimed to enhance the understanding of CBD consumption patterns, specifically exploring potential factors influencing this pattern: health conditions, with a focus on psychiatric conditions, prescribed medication use, including psychotropic medication, medical supervision, sex, and age. Additionally, we were particularly interested in the role of cannabis use as a potential factor affecting CBD usage patterns. We hypothesized that these variables will influence the pattern of CBD product use, including dosage, perceived effectiveness, side effects, route of administration, and duration of use.

The increasing use of CBD as a food supplement and its presence in a diverse range of products raises questions about consumer behavior, motivations, and the perceived efficacy of these products. This aspect is particularly pertinent given the concerns raised by Kirkland et al. (16) regarding the current lack of high-quality evidence supporting CBD use for psychiatric disorders. Furthermore, discrepancies in product content and labeling (19, 20) underscore the necessity for research that not only investigates the biochemical efficacy of CBD but also considers the consumer experience and regulatory landscape. By examining these factors, our study seeks to contribute to a more holistic understanding of CBD’s role in health and wellness. In sum, this study is not merely an exploration of CBD’s therapeutic potential; it is a comprehensive examination of how CBD is being integrated into people’s lives, addressing a critical need for empirical data to guide future research, policy-making, and informed decision-making by consumers and healthcare providers.

## 2 Methods

An anonymous online questionnaire was developed to gather self-reported information on CBD usage characteristics. The survey was deployed on the Internet using Google Forms, Google’s online survey tool, with data collected from August 2021 to February 2022. Distribution occurs via various social media channels.

In our study, CBD products were defined as all commercial products available on the market without a prescription containing cannabidiol (CBD) with a THC content of no more than 0.2% (legally available in Poland at the time of the study; however, new legislation has changed to less than 0.3% after the study was completed), such as oil, flower, and cosmetic products. Cannabis was defined as cannabis products with a higher THC content exceeding 0.2% THC, often referred to as marijuana.

### 2.1 Survey eligibility criteria

To qualify for survey completion, the respondents were required to be ≥18 years old. Upon providing consent, the participants encountered an initial demographic question and a screening question regarding previous CBD product usage. If the response was “No,” no further inquiries were made. For those who responded affirmatively, a complete section of the questions was presented.

### 2.2 Content of the questionnaire

The questionnaire included self-reported demographic details (age and sex), CBD usage patterns (frequency, dosage, purpose, duration, perceived effectiveness, and side effects), cannabis use, and health conditions (diagnosed mental disorders, psychotropic or prescribed medication usage, and being under medical supervision). The full questionnaire is provided in the **Supplementary Materials**. Personal data and IP addresses were not collected. Ethical approval was deemed unnecessary, as the research involved non-sensitive information, utilized anonymous survey methods, and involved participants not categorized as “vulnerable.” Additionally, participation was not expected to induce undue psychological stress or anxiety.

### 2.3 Statistical analyses

R software, version 2022.07.2, was used for statistical analyses, with the significance set at p < 0.05. The χ2-test, along with Cramer’s V (considering 0.3 as a medium effect and 0.5 as a large effect), assessed categorical variables’ relationships, while t-tests examined continuous variables (cannabis use and age). Spearman’s rho (rs) and point biserial correlation (rpb) gauged correlations between continuous and dichotomous variables, respectively.

To compute correlations involving daily CBD dosage and other variables (dichotomous or continuous), responses indicating “I don’t know” were excluded from analyses. Although ordinal CBD usage data are presented in **Table 2** as percentages, they were coded numerically in the correlation analyses.

## 3 Results

### 3.1 Study sample population

A total of 334 participants completed the survey (M = 88; F = 246), with a mean age of 30.28 (SD = 7.53, MIN = 18, MAX = 57). Only four participants (1.2%) reported unfamiliarity with CBD, while 67 (20.1%) acknowledged that CBD was not used (M = 4; F = 63; age = 30.57, SD = 8.72). Only participants who used CBD were included in the study and subsequent analyses (n = 267). Among them, 25.8% (n = 69) reported diagnosed psychiatric disorders, 21.3% (n = 57) used psychotropic medications, and 39% (n = 104) were under medical supervision for health conditions. Nearly half of the sample (46.8%, n = 125) admitted to using prescribed medication, and 57.3% (n = 153) were actively using CBD products at the time of the survey. Cannabis use was reported by 132 (49.4%) respondents included in the study.

Compared with individuals without psychiatric diagnoses, individuals with psychiatric diagnoses were more likely to be younger. Individuals with psychiatric diagnoses were significantly more likely to be under medical supervision due to health conditions and to use prescribed and psychotropic medication than individuals without psychiatric diagnoses. Both groups did not differ in the case of sex and cannabis use. Detailed information for each group and statistics are presented in **Table 1**.

**Table 1:** Demographic characteristics of CBD users with and without diagnosed psychiatric disorders.

	All participants (n= 267)	Participants with diagnosed psychiatric disorders (n=69)	Participants without diagnosed psychiatric disorders (n=198)	Statistics
Age in years M (SD)	30.21 (7.21)	28.7 (6.25)	30.7 (7.46)	(t(140.3) = 2.18, p = 0.031
Female Sex	183 (68.5%)	52 (75.4%)	131 (66.2%)	χ2 = 1.61, p = 0.21, Cramer V = 0.08
Psychotropic medication	57 (21.3%)	48 (69.6%)	9 (4.6%)	χ2 = 125, p < 0.001, Cramer V = 0.69
Cannabis use	132 (49.4%)	36 (52.2%)	96 (48.5%)	χ2 = 0.15, p = 0.7, Cramer V = 0.02
Prescribed medication	125 (46.8%)	51 (73.9%)	74 (37.4%)	χ2 = 26, p < 0.001, Cramer V = 0.31
Under medical supervision due to health condition	104 (39%)	56 (81.2%)	48 (24.2%)	χ2 = 67.34, p < 0.001, Cramer V = 0.5

Compared with cannabis non-users, cannabis users were more likely to be younger and male. Cannabis non-users were significantly more likely to be under medical supervision due to health conditions than cannabis users, but did not differ from cannabis users in the use of psychotropics, any prescribed medication use, or diagnosed psychiatric disorders. Detailed information for each group and statistics are presented in **Table 2**.

**Table 2:** Demographic characteristics of CBD users with and without cannabis use.

	All participants (n= 267)	Cannabis users (n=132)	Cannabis non-users (n=135)	Statistics
Age in years M (SD)	30.21 (7.21)	28.59 (6.24)	31.8 (7.75)	t(255.63) = 3.73, p < 0.001
Female Sex	183 (68.5%)	69 (52.3%)	114 (84.4%)	χ2 = 30.56, p < 0.001, Cramer V = 0.35
Diagnosed psychiatric disorders	69 (25.8%)	36 (27.3%)	33 (24.4%)	χ2 = 0.156, p = 0.698, Cramer V = 0.03
Psychotropic medication	57 (21.3%)	27 (20%)	30 (22.7%)	χ2 = 0.16, p = 0.693, Cramer V = 0.03
Prescribed medication	125 (46.8%)	70 (51.9%)	55 (41.7%)	χ2 = 2.37, p = 0.122, Cramer V = 0.1
Under medical supervision due to health condition	104 (39%)	42 (31.8%)	62 (45.9%)	χ2 = 5.01, p = 0.025, Cramer V = 0.14

### 3.2 Length of use

The majority of participants (71.2%) reported using CBD for up to 6 months, with the highest prevalence within 0-3 months (48.7%). The data showed that 57.3% of the entire sample reported currently using CBD products.

There was no significant relationship between the length of CBD use and psychiatric disorders, psychotropic medication, or any prescribed medication use nor medical supervision (see **Table 3**).

**Table 3:** Patterns of cannabidiol use in participants with and without diagnosed psychiatric disorders.

	All participants (n= 267)	Participants with diagnosed psychiatric disorders (n=69)	Participants without diagnosed psychiatric disorders (n=198)	Statistics
**Length of CBD use**				**r = -0.004, p = 0.95; point biserial correlation**
0–3 months	130 (48.69%)	36 (52.2%)	94 (47.5%)	
3–6 months	60 (22.47%)	11 (15.9%)	49 (24.8%)	
6–12 months	34 (12.73%)	11 (15.9%)	23 (11.6%)	
1-2 years	25 (9.36%)	7 (10.1%)	18 (9.1%)	
2-5 years	16 (5.99%)	3 (4.4%)	13 (6.6%)	
5 years <	2 (0.75%)	1 (1.5%)	1 (0.5%)	
**Route of administration**				
Vaping	51 (19.1%)	13 (18.8%)	38 (19.2%)	χ2 <0.01, p=1, Cramer V = 0.003
Smoking	100 (37.45%)	29 (42%)	71 (35.9%)	χ2 = 0.59, p = 0.44, Cramer V = 0.06
Sublingual	196 (73.41%)	51 (73.9%)	145 (73.2%)	χ2 <0.01, p=1, Cramer V = 0.04
Capsules/pills	19 (7.12%)	4 (5.8%)	15 (7.6%)	
Topical (on skin)	25 (9.36%)	19 (9.6%)	5 (7.3%)	
Edibles	30 (11.24%)	8 (11.6%)	2 (10.1%)	
Spray (via mouth)	2 (0.75%)	1 (1.5%)	1 (0.5%)	
Drinking	4 (1.50%)	2 (2.9%)	2 (1%)	
Suppository (rectal)	1 (0.37%)	1 (0.51)	0	
**Daytime use**				
Morning	21 (7.87%)	5 (7.3%)	16 (8.1%)	
Evening	93 (34.83%)	21 (30.4%)	72 (36.4%)	
Morning and evening	53 (19.85%)	11 (15.9%)	42 (21.2%)	
Few times per day (> 2)	40 (14.98%)	11 (15.9%)	29 (14.7%)	
When needed	60 (22.47%)	21 (30.4%)	39 (19.7%)	
**Daily dosage (mg)**				**r= 0.13, p = 0.09; point biserial correlation**
0–24	72 (27.1%)	12 (17.4%)	60 (30.5%)	
25–49	45 (16.9%)	10 (14.5%)	35 (17.8%)	
50–99	32 (12.1%)	8 (11.6%)	24 (12.2%)	
100–149	4 (1.5%)	2 (2.9%)	2 (1%)	
150–199	4 (1.5%)	1 (1.5%)	3 (1.5%)	
≥ 200	12 (4.5%)	4 (5.8%)	8 (4.1%)	
I don’t know	97 (36.5%)	32 (46.4%)	65 (33%)	
Perceived effectiveness	182 (68.2%)	40 (57.8%)	142 (71.7%)	χ2 = 3.84, p = 0.0499, Cramer V = 0.13
Currently using CBD products	153 (57.3%)	34 (49.3%)	119 (60.1%)	
Perceived side effects	30 (11.2%)	7 (10.29%)	23 (11.7%)	χ2 = 0.01, p=0.92, Cramer V = 0.02
**Top reasons for using CBD**				
Stress	173 (65.3%)	52 (76.5%)	121 (61.4%)	χ2 = 4.41, p = 0.036, Cramer V = 0.14
Sleep	138 (51.7%)	35 (50.7%)	103 (52%)	χ2 = 0.002, p = 0.96
Overall well-being	139 (52.5%)	36 (52.9%)	103 (52.3%)	χ2 <0.01, p=1, Cramer V = 0.006
Mood	120 (44.9%)	36 (52.2%)	84 (42.4%)	χ2 = 1.59, p = 0.21, Cramer V = 0.01
Anxiety	109 (40.9%)	45 (65.2%)	64 (32.2%)	χ2 = 21.6, p< 0.0001, Cramer V = 0.3

Cannabis users reported using CBD products for a longer duration than non-users (see **Table 4**).

**Table 4:** Patterns of cannabidiol use in cannabis users and non-users.

	All participants (n= 267)	Cannabis users (n=132)	Cannabis non-users (n=135)	Statistics
Length of CBD use				r = 0.18, p = 0.004; point-biserial correlation
0–3 months	130 (48.69%)	56 (42.4%)	74 (54.8%)	
3–6 months	60 (22.47%)	29 (22%)	31 (23%)	
6–12 months	34 (12.73%)	18 (16.3%)	16 (11.9%)	
1-2 years	25 (9.36%)	16 (12.1%)	9 (6.7%)	
2-5 years	16 (5.99%)	11 (8.3%)	5 (3.7%)	
5 years <	2 (0.75%)	2 (1.5%)	–	
Route of administration				
Vaping	51 (19.1%)	45 (34.09%)	6 (4.44%)	χ2 = 36.07, p <0.0001, Cramer V = 0.38
Smoking	100 (37.45%)	85 (64.39%)	15 (11.15%)	χ2 = 78.63, p <0.0001, Cramer V = 0.55
Sublingual	196 (73.41%)	80 (60.61%)	116 (85.93%)	χ2 = 22.11, p < 0.0001, Cramer V = 0.3
Capsules/pills	19 (7.12%)	10 (7.58%)	9 (6.67%)	
Topical (on skin)	25 (9.36%)	16 (12.12%)	9 (6.67%)	
Edibles	30 (11.24%)	20 (15.15%)	10 (7.41%)	
Spray (via mouth)	2 (0.75%)	0	2 (1.48%)	
Drinking	4 (1.50%)	1 (0.76%)	3 (2.22%)	
Suppository (rectal)	1 (0.37%)	0	1 (0.74%)	
Daytime use				
Morning	21 (7.87%)	4 (3%)	17 (12.6%)	
Evening	93 (34.83%)	49 (37.1%)	44 (32.6%)	
Morning and evening	53 (19.85%)	18 (13.6%)	35 (25.9%)	
Few times per day (> 2)	40 (14.98%)	20 (15.2%)	20 (14.8%)	
When needed	60 (22.47%)	41 (31.1%)	19 (14.1%)	
Daily dosage (mg)				r= -0.11, p = 0.14; point biserial correlation
0–24	72 (27.1%)	33 (25%)	39 (28.9%)	
25–49	45 (16.9%)	22 (16.7%)	23 (17%)	
50–99	32 (12.1%)	16 (12.1%)	16 (11.9%)	
100–149	4 (1.5%)	4 (3%)	–	
150–199	4 (1.5%)	–	4 (3%)	
≥ 200	12 (4.5%)	2 (1.5%)	10 (7.4%)	
I don’t know	97 (36.5%)	54 (40.9%)	43 (31.9%)	
**Perceived effectiveness**	182 (68.2%)	99 (75%)	83 (61.5%)	χ2 = 5.02, p = 0.025, Cramer’s V = 0.15
**Currently using CBD products**	153 (57.3%)	77 (58.3%)	76 (56.3%)	
**Perceived side effects**	30 (11.2%)	8 (6.1%)	22 (16.3%)	χ2 = 6.14, p = 0.013, Cramer V = 0.16
Top reasons for using CBD				
Stress	173 (65.3%)	86 (65.7%)	87 (64.9%)	χ2 <0.01, p = 1, Cramer V = 0.007
Sleep	138 (51.7%)	68 (51.2%)	70 (51.9%)	χ2 <0.01, p = 1, Cramer V = 0.003
Overall well-being	139 (52.5%)	74 (56.1%)	65 (48.9%)	χ2 = 1.1, p = 0.29, Cramer V = 0.07
Mood	120 (44.9%)	66 (50%)	54 (40%)	χ2 = 2.31, p = 0.13, Cramer V = 0.1
Anxiety	109 (40.9%)	47 (35.6%)	62 (45.9%)	χ2 = 2.53, p = 0.11, Cramer V = 0.1

Males tended to use CBD products for a longer duration than females. There was no significant relationship between the length of CBD use and age (see **Table 1A** in **Supplementary Materials**).

### 3.3 Route of administration

The most common route of CBD administration reported by participants was sublingual (73.41%), followed by smoking (37.45%), and vaping (19.1%), with more detailed analyses conducted.

There were no significant differences in diagnosed psychiatric disorders and sublingual CBD use, by smoking, or vaping. Moreover, there was no significant difference in medical supervision and sublingual CBD use, by smoking, and vaping. There were no significant differences in the use of psychotropic medication and CBD sublingually, by smoking, or vaping. Those using prescribed medication were more likely than those who were not to sublingually administer CBD (80% vs. 68.31%), whereas those not using prescribed medication were more likely than those who were using to administer CBD via smoking (62% vs. 38%). There was no significant difference between the use of prescribed medications and CBD via vaping (see **Table 3**; **Table 1A** in **Supplementary Materials**).

Cannabis non-users were more likely than cannabis users to use CBD sublingually, while cannabis users were more likely than non-users to use CBD via smoking, and vaping (see **Table 4**).

Females were more likely than males to use CBD sublingually (79.78% vs. 60.71%), while males were more likely than females to use CBD by smoking (60.71% vs. 26.78%) and vaping (38.2% vs. 10.38%). There was a significant correlation between age and smoking and the sublingual route of administration, but not with vaping. Older individuals preferred to consume CBD sublingually, whereas younger individuals preferred to smoke (see **Table 1A** in **Supplementary Materials**).

### 3.4 Time of consumption and daily dosage

The majority of participants in this study reported using CBD products in the evening (34.83%), when needed (22.47%), and twice a day: morning and evening (19.85%).

The data on daily CBD dosage showed that the majority of participants (36.5%) reported not knowing their daily dosage. Among those who reported their dosage, the most common range was 0-24 mg (27.1%), followed by 25-49 mg (16.9%) and 50-99 mg (12.1%). There was no significant relationship between daily CBD dosage and diagnosed psychiatric disorder, or medical supervision. Respondents who reported using psychotropic medication consumed a higher daily CBD dosage, as well as those who reported any prescribed medication use (see **Table 3**; **Table 1A** in **Supplementary Materials**).

There was no significant relationship between daily CBD dosage and cannabis use (see **Table 4**).

Moreover, there was no significant relationship between daily CBD dosage and sex or age (**Table 1A** in **Supplementary Materials**).

### 3.5 Reasons for using CBD

**Figure 1:**
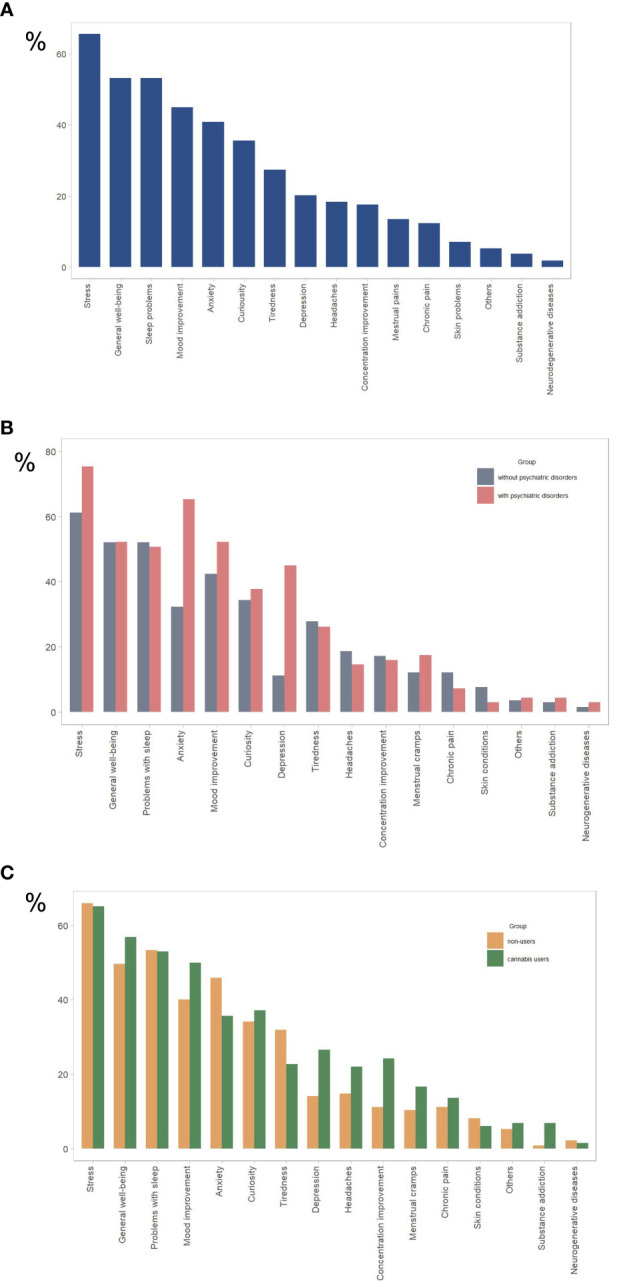
Reasons for using CBD products for **(A)** all study participants (n= 267), **(B)** participants with psychiatric disorders (n = 69) and without psychiatric disorders (n = 198), **(C)** cannabis users (n=132), and cannabis nonusers (n=135). The y-axis represents the percentage of total responses for each group (N). Participants were allowed to select multiple options.

Among the total sample, the most common reasons for using CBD products were stress (65.3%), sleep problems (51.7%), overall well-being improvement (52.5%), mood improvement (44.9%), and anxiety (40.9%) (**Figure 1A**). These data align with those of other research on the reasons for using CBD products (13, 21). Other reasons included curiosity, tiredness, depression, chronic and menstrual pain, skin problems, and neurodegenerative diseases.

Individuals with psychiatric conditions were significantly more likely than those without to use CBD to relieve stress (76.5% vs. 61.4%) and anxiety (65.2% vs. 32.3%), but not to improve sleep quality), mood, or overall well-being (**Table 3**; **Figure 1B**). Individuals using psychotropic medication were significantly more likely than those not using it to use CBD to relieve stress (81.8% vs 70%) and anxiety (70.2% vs 32.9%), but not to improve sleep quality, mood, or overall wellbeing. Individuals using any prescribed medication were significantly more likely than those not using to use CBD to relieve anxiety (48.8% vs. 33.8%), but not to improve sleep quality, mood, overall well-being, or relieve stress (see **Table 3**; **Table 1A** in **Supplementary Materials**).

Individuals under medical supervision were significantly more likely than those not to use CBD to relieve anxiety (51% vs. 34.4%)), but not stress, improve sleep quality, mood, or overall well-being (**Table 1A** in **Supplementary Materials**).

There was no significant difference between cannabis users and non-users in the use of CBD products to relieve stress and anxiety and improve sleep, well-being, or mood (**Table 4**; **Figure 1C**).

Males were more likely than females to use CBD products to relieve anxiety (54.6% vs. 45.4%). There were no significant differences between females and males in using CBD products for stress relief, sleep quality, overall well-being, or mood improvement. There was no significant correlation between age and the use of CBD to improve mood, sleep, relieve anxiety or stress. A significant correlation was observed between age and CBD use to improve well-being. Younger individuals tend to use CBD to improve their wellbeing (**Table 1A** in **Supplementary Materials**).

### 3.6 Effectiveness

Among the total sample, 68.2% reported CBD products as effective to relieve their symptoms.

Individuals without psychiatric conditions were significantly more likely than those with to admit that CBD was effective in relieving their symptoms (71.7% vs. 57.8%, **Table 3**). There was no significant difference in perceived CBD effectiveness between those using and not using psychiatric medication, those under medical supervision, or those using any prescribed medication (**Table 1A** in **Supplementary Materials**).

Cannabis users were significantly more likely than non-users to admit that CBD was effective in relieving their symptoms (75% vs.61.5%, **Table 4**).

There was no significant difference in CBD effectiveness between males and females, and there was no significant correlation between age and CBD effectiveness (**Table 1A** in **Supplementary Materials**).

### 3.7 Side effects

**Figure 2:**
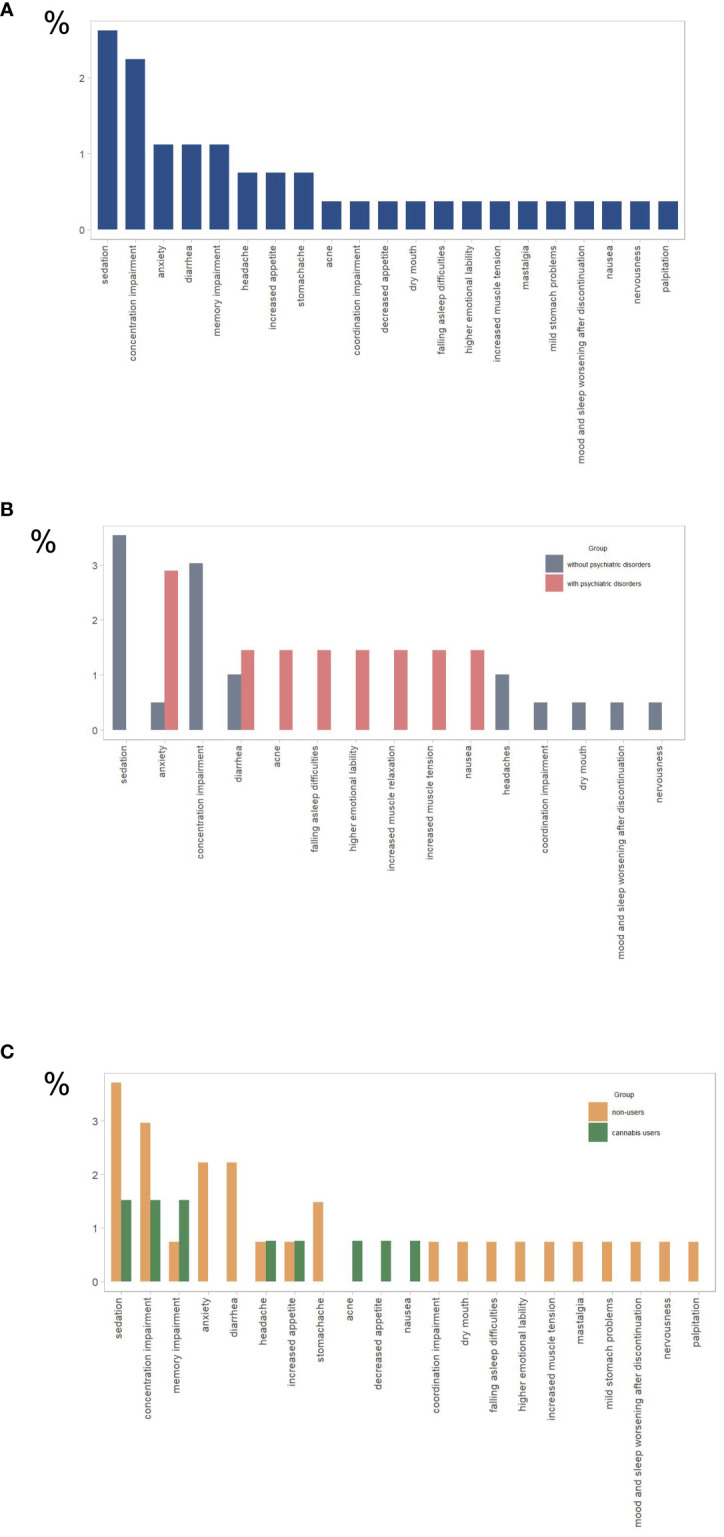
Reported side effects for **(A)** all study participants (n= 267), **(B)** participants with psychiatric disorders (n = 69) and without psychiatric disorders (n = 198), **(C)** cannabis users (n=132), and cannabis nonusers (n=135). The y-axis represents the percentage of total responses for each group (N).

Of the total sample, 11.2% reported experiencing side effects related to CBD product usage, with more detailed information shown in **Figure 2A**.

There were no significant differences in the reporting of side effects among individuals with diagnosed psychiatric conditions, those using psychotropic medication or any prescribed medication, or those under medical supervision compared to those without such conditions (see **Figure 2B** and **Table 3**; **Table 1A** in **Supplementary Materials**).

Cannabis non-users were significantly more likely than cannabis users to experience CBD side effects (16.3% vs. 6.1%), as shown in **Figure 2C** (**Table 4**).

There were no significant differences in the reporting of side effects between males and females. A significant correlation was observed between age and CBD side effects (**Table 1A** in **Supplementary Materials**). Older individuals tend to report side effects more often.

## 4 Discussion

This study represents the inaugural exploration of CBD use patterns with a focus on health conditions, mainly distinguishing between individuals with diagnosed mental disorders and not, but also cannabis users and non-users.

The top reasons for CBD use in our study are generally consistent with those of other cross-sectional studies of CBD use patterns, as the majority of participants reported using CBD to relieve stress and anxiety, improve sleep, mood, and overall well-being (12–14). Moreover, our results suggest that individuals with psychiatric conditions and those using psychotropic medication were more likely to use CBD products to relieve stress and anxiety (medium size effect). Furthermore, individuals under medical supervision and those using prescribed medication were more likely to use CBD products to relieve anxiety, which may suggest that these individuals are seeking additional support for their mental health concerns beyond their current treatment regimen. This information may be useful for healthcare providers working with these patient populations to develop targeted interventions to address these symptoms and provide evidence-based education about CBD, including potential drug interactions. Another cross-sectional study particularly focused on CBD in self-treatment of depression has shown only about half of the group of psychiatric patients informed their psychiatrist about CBD use (22).

Research on CBD’s potential to treat anxiety disorders has shown promising results, with small randomized controlled trials indicating its anxiolytic effects (23). The exact mechanisms underlying the impact of CBD on the body remain unclear; however, they interact with serotonin receptors and modulate CB1 receptor activation, potentially influencing anxiety-related brain structures (4, 24). The results regarding the effects of CBD on sleep are inconsistent, with studies showing varied outcomes (25, 26, preprint; 27, 28). Notably, lower CBD doses (18–25 mg) demonstrated positive effects on anxiety and sleep in retrospective studies and individual patient case reports (29, 30).

Preclinical studies have indicated the potential of CBD as a fast-acting and sustained antidepressant that induces neuroplastic alterations in brain structures associated with depression (7–9). However, evidence from human studies that support the mood-improving effects of CBD in patients with depression is insufficient.

Despite these potential benefits, our study highlights the importance of considering factors beyond dosage adjustment to fully optimize CBD treatment for anxiety, sleep, and depression.

Moltke and Hindocha’s (13) observational study revealed that the most common pattern of CBD product use for stress, anxiety, and sleep issues involved a daily dose below 50 mg. However, approximately 17% reported exceeding 100 mg daily, and approximately 10% were uncertain about their CBD dosage. Our study mirrored these trends, with over 40% reporting daily dosages below 50 mg but around 20% surpassing 50 mg. Intriguingly, approximately 36% of participants were unsure of their dosage, underscoring the need for enhanced education and customer information. Notably, individuals taking psychotropic medication reported higher daily CBD dosages, similar to those using prescribed medication. Evening has emerged as the most common time for CBD use, which is consistent with prior studies (12, 13).

Approximately 70% of the participants perceived CBD products as effective, while individuals without psychiatric conditions reported higher perceived effectiveness than those with psychiatric conditions. The variation in perceived effectiveness between individuals with and without psychiatric conditions may be attributed to several factors. Individuals without psychiatric conditions may experience fewer pre-existing symptoms that could potentially mask the effects of CBD products. Additionally, the psychological state of individuals with psychiatric conditions might influence their perception of product efficacy. Additional research is necessary to ascertain the efficacy of CBD products and dosing regimens for diverse psychiatric conditions. It is crucial to recognize that perceived effectiveness may not always correlate with objective effectiveness.

Moreover, a higher perceived effectiveness of CBD was observed among cannabis users compared to non-users. This discrepancy suggests that prior cannabis use may influence CBD product perception. Further research is imperative to validate this finding and to explore the potential underlying reasons.

Clinical studies affirm the safety and tolerability of CBD, with doses up to 6000 mg showing no serious effects (31, 32). The most common side effects were gastrointestinal symptoms, somnolence, and loss of appetite, but they were not severe (33). In our study, overall about 10% of individuals reported experiencing side effects of which the most frequently reported were concentration and memory impairment, sedation, anxiety, and diarrhea. Interestingly, side effects were more common in cannabis non-users than in cannabis user groups as well as in older individuals. Some side effects such as concentration and memory impairment or anxiety could most likely be indicating levels of THC in the product, as these are common side effects of THC (e.g. 5, 34), especially in cannabis non-users. It is important to note that the CBD used for research has a safe profile, but CBD sold in various commercial products is not always safe. In a published study that analyzed the composition of CBD extracts ordered online, it turned out that about 70% were off-label (with higher or lower CBD and/or THC ratio) (35). Moreover, pesticides, mold, lead, and other substances, including synthetic cannabinoids, have been detected in such products (36, 37). CBD’s potential interactions with commonly used drugs warrant further consideration (38).

Our findings indicate a striking similarity in CBD usage patterns between groups, with some notable distinctions in the routes of administration of CBD products. We have not observed any differences related to psychiatric conditions. However, sublingual use was more favored among participants using prescribed medication, also smoking was less popular in this group. Moreover, sublingual use was more favored among cannabis non-users, while cannabis users lean towards smoking and vaping (with medium to large effect sizes). This may be attributed to cannabis users’ familiarity with inhalation methods, reminiscent of those popular in cannabis consumption (39), and their preference for immediate effects. In contrast, people using prescribed medication and cannabis non-users may prioritize health consciousness and seek discreet or convenient administration methods. Similar trends were noted among females and older individuals.

Notably, sublingual, smoking and vaping methods have emerged as the most prevalent. This aligns with existing research that emphasizes the prevalence of sublingual administration in CBD product usage (13, 14).

This study has several important limitations that should be taken into account when interpreting the results. One potential limitation is that the data were self-reported by participants, which may introduce recall bias or social desirability bias. This could impact the accuracy and reliability of the findings, particularly regarding sensitive topics such as cannabis use. Additionally, the study did not collect data on the frequency, product kind, or dosage of cannabis use, as well as usage onset which may have an important impact on the observed effects. Moreover, the study did not capture specific details about the types or formulations of CBD products used, which could influence outcomes.

Another limitation of this study is that the effect sizes were generally small, and the sample size was modest. This may limit the generalizability of the findings to other populations or contexts. Furthermore, the sample was not randomized, and participants were recruited through social media platforms, which may have resulted in self-selection bias and overrepresentation of certain subgroups.

Furthermore, the limitations extend to the lack of nuanced exploration regarding participants’ motivations for CBD use and how it may contribute to improvements in their life quality. This oversight hinders a comprehensive understanding of the multifaceted factors influencing participants’ perceptions and experiences.

Finally, the study included a limited number of individuals with psychiatric comorbidities, which could impact the generalizability of findings to populations with diverse health conditions. The absence of a more comprehensive representation of individuals with various comorbidities limits the applicability of the results to broader health contexts. In summary, the limitations outlined, including but not limited to those mentioned, underscore the need for caution in extrapolating the findings and emphasize areas for improvement in future research.

A significant strength of this study was its emphasis on health conditions, particularly the differentiation between individuals diagnosed with mental disorders and those without, as well as between cannabis users and non-users, in exploring CBD usage patterns. While the findings offer valuable insights into the behaviors of these specific populations regarding CBD usage, it’s essential to consider the limitations outlined previously.

Implications of this study extend to several areas. Firstly, healthcare providers should be aware of the prevalent use of CBD among individuals with psychiatric conditions and those using psychotropic medication, particularly for stress and anxiety relief. This highlights the importance of open communication between patients and clinicians regarding CBD supplementation, as well as the need for targeted interventions to address symptoms and provide evidence-based education about CBD.

Secondly, the variability in the perceived effectiveness of CBD products among different population groups underscores the necessity for further research to ascertain the efficacy of CBD for diverse psychiatric conditions and dosing regimens. Additionally, the observed discrepancy in perceived effectiveness between cannabis users and non-users warrants exploration into potential underlying reasons, which could inform personalized treatment approaches.

Furthermore, the findings regarding CBD dosage patterns and safety profiles emphasize the importance of enhanced education and consumer guidance in the CBD market. Healthcare providers should remain vigilant for potential adverse effects, especially among cannabis non-users and older individuals, and consider the potential interactions with other factors or compounds present in CBD products.

Lastly, the study’s limitations highlight the need for future research endeavors to address these gaps. Larger, randomized studies with comprehensive data collection on cannabis use patterns, CBD product types, dosages, and motivations for use are necessary to provide a more nuanced understanding of CBD’s role in mental health management.

## 5 Conclusion

CBD has emerged as a promising tool for managing prevalent health issues, notably stress, anxiety, depressed mood, and sleep disorders. Many individuals attest to its efficacy in treating these conditions, without severe side effects. These findings underscore the need for further investigation of the therapeutic potential of CBD across a spectrum of medical conditions, addressing concerns surrounding safety, effectiveness, and optimal dosing.

Our study highlights the importance of considering specific health factors, including psychiatric conditions, prescribed medications (especially psychotropic medications), medical supervision, and cannabis use, for a more nuanced understanding of CBD usage patterns. This holistic approach enables a comprehensive exploration of the influence of various health variables on CBD consumption.

Ongoing research and education are imperative in the dynamic landscape of CBD use. Both patients and healthcare providers require reliable information to navigate the potential benefits and risks associated with CBD products. By staying informed and conducting thorough research, we can pave the way for more informed and responsible use of CBD in diverse medical contexts.

## Data availability statement

The raw data supporting the conclusions of this article will be made available by the authors, without undue reservation.

## Ethics statement

Ethical approval was not required since this research investigated non-sensitive information using completely anonymous survey procedures when the participants are not defined as “vulnerable” and participation will not induce undue psychological stress or anxiety. The participants consented to participate by answering the online survey. The studies were conducted in accordance with the local legislation and institutional requirements. Written informed consent for participation was not required from the participants or the participants’ legal guardians/next of kin in accordance with the national legislation and institutional requirements because the research involved non-sensitive information, utilized anonymous online survey methods, and involved participants not categorized as “vulnerable.” Additionally, participation was not expected to induce undue psychological stress or anxiety. Personal data and IP addresses were not collected.

## Author contributions

AAB: Conceptualization, Formal analysis, Investigation, Methodology, Project administration, Validation, Writing – original draft, Writing – review & editing. NJ: Visualization, Writing – original draft, Writing – review & editing. AR: Writing – original draft, Writing – review & editing. SL: Writing – original draft, Writing – review & editing. SS: Writing – original draft, Writing – review & editing. AB: Writing – original draft, Writing – review & editing.
