# Supplementary material for: Cannabidiol usage, efficacy, and side effects: analyzing the impact of health conditions, medications, and cannabis use in a cross-sectional online pilot study
Source: Front Psychiatry. 2024 Feb 29;15:1356009. doi: 10.3389/fpsyt.2024.1356009 (PMC10938386; doi:10.3389/fpsyt.2024.1356009)
Supplement: Supplementary file 1 [file Table_1.docx]

Table 1A. Results of statistical analysis.

|  | Groups/variables compared | Statistics |
| --- | --- | --- |
| Length of CBD use | Sex | r = -0.19a, p = 0.002* |
|  | Age | r = 0.06b, p = 0.34 |
|  | Psychotropic medication use | r = 0.006a, p = 0.92 |
|  | Prescribed medication use | r = 0.008a, p = 0.9 |
|  | Under medical supervision due to health condition | r = 0.02a, p = 0.76 |
| Route of administration | | |
| Sublingual | Sex | χ2 = 9.86, p = 0.002*, Cramer V = 0.22 |
|  | Age | r = 0.19a, p= 0.002* |
|  | Psychotropic medication use | χ2 = 1.37, p = 0.24, Cramer V = 0.09 |
|  | Prescribed medication use | χ2 = 4.11, p = 0.043*, Cramer V = 0.15 |
|  | Under medical supervision due to health condition | χ2 = 1.15, p = 0.28, Cramer V = 0.1 |
| Smoking | Sex | χ2 = 26.88, p < 0.0001*, Cramer V = 0.33 |
|  | Age | r = -0.21a, p=0.0006* |
|  | Psychotropic medication use | χ2 = 0.07, p = 0.79, Cramer V = 0.03 |
|  | Prescribed medication use | χ2 = 4.44, p = 0.035*, Cramer V = 0.14 |
|  | Under medical supervision due to health condition | χ2 = 3.73, p = 0.053, Cramer V = 0.13 |
| Vaping | Sex | χ2 = 26.84, p < 0.0001*, Cramer V = 0.33 |
|  | Age | r = -0.11a, p=0.07 |
|  | Psychotropic medication use | χ2 = 0.022, p = 0.88, Cramer V = 0.02 |
|  | Prescribed medication use | χ2 = 2.81, p = 0.093, Cramer V = 0.11 |
|  | Under medical supervision due to health condition | χ2 = 0.014, p = 0.91, Cramer V = 0.02 |
| Daily CBD dosage | Sex | r= -0.027a, p = 0.72 |
|  | Age | r= 0.05b, p = 0.49 |
|  | Psychotropic medication use | r= 0.23a, p =0.002* |
|  | Prescribed medication use | r= 0.17a, p = 0.03* |
|  | Under medical supervision due to health condition | r= 0.11a, p = 0.16 |
| Perceived effectiveness | Sex | χ2 = 0.05, p = 0.83, Cramer V = 0.02 |
|  | Age | r= -0.06a, p = 0.3 |
|  | Psychotropic medication use | χ2 = 1.16, p = 0.28, Cramer V = 0.08 |
|  | Prescribed medication use | χ2 = 0.2, p = 0.65, Cramer V = 0.04 |
|  | Under medical supervision due to health condition | χ2 = 0.14, p = 0.71, Cramer V = 0.03 |
| Perceived side effects | Sex | χ2 = 0.51, p = 0.47, Cramer V = 0.06 |
|  | Age | r = 0.17b, p = 0.005* |
|  | Psychotropic medication use | χ2 <0.01, p=1, Cramer V = 0.01 |
|  | Prescribed medication use | χ2 = 0.93, p = 0.34, Cramer V = 0.07 |
|  | Under medical supervision due to health condition | χ2 = 0.19, p = 0.66, Cramer V = 0.04 |
| Top reasons for using CBD | | |
| Stress | Sex | χ2 = 2.19, p = 0.14, Cramer V = 0.09 |
|  | Age | r = -0.07a, p = 0.25 |
|  | Psychotropic medication use | χ2 = 7.48,p = 0.006*, Cramer V = 0.18 |
|  | Prescribed medication use | χ2 = 1.81, p = 0.18, Cramer V = 0.09 |
|  | Under medical supervision due to health condition | χ2 = 1.94, p = 0.16, Cramer V = 0.09 |
| Anxiety | Sex | χ2 = 4.37, p = 0.037*, Cramer V = 0.14 |
|  | Age | r = -0.05a, p = 0.44 |
|  | Psychotropic medication use | χ2 = 24.32, p< 0.0001*, Cramer V = 0.31 |
|  | Prescribed medication use | χ2 = 5.58, p = 0.018*, Cramer V = 0.15 |
| Currently using CBD products | Under medical supervision due to health condition | χ2 = 6.58, p = 0.01*, Cramer V = 0.16 |
| Sleep | Sex | χ2 = 1.68, p = 0.19, Cramer V = 0.09 |
|  | Age | r = -0.05a, p = 0.4 |
|  | Psychotropic medication use | χ2 = 0.37,p = 0.54, Cramer V = 0.05 |
|  | Prescribed medication use | χ2 = 0.91, p = 0.34, Cramer V = 0.07 |
|  | Under medical supervision due to health condition | χ2 = 0.19, p = 0.66, Cramer V = 0.03 |
| Mood | Sex | χ2 = 1.58, p = 0.21, Cramer V = 0.09 |
|  | Age | r = -0.06a, p = 0.37 |
|  | psychotropic medication use | χ2 = 0.32, p = 0.57, Cramer V = 0.04) |
|  | prescribed medication use | χ2 = 0.028, p = 0.87, Cramer V = 0.02 |
|  | medical supervision | χ2 = 0.004, p = 0.95, Cramer V = 0.01 |
| Overall wellbeing | Sex | χ2 = 0.62, p = 0.43, Cramer V = 0.06 |
|  | Age | r =-0.16a, p = 0.008* |
|  | psychotropic medication use | χ2 = 0.18, p = 0.68, Cramer V = 0.03 |
|  | prescribed medication use | χ2 = 0.37,p = 0.54, Cramer V = 0.04 |
|  | medical supervision | χ2 = 0.39, p = 0.53, Cramer V = 0.05 |

Note: a point biserial correlation; bSpearman's rank correlation rho*Significant results p < 0.05

User Survey

1. Gender

- Male
- Female

1. Age
2. Have you heard about CBD products?

- Yes
- No

1. Have you used CBD products?

- Yes
- No

1. Do you stay under medical supervision due to chronic health condition?

- Yes
- No

1. Do you suffer from a mental disorder diagnosed by a psychiatrist?

- Yes
- No

1. Are you currently taking any prescribed medications?

- Yes
- No

1. Are you taking medications for sleep, sedatives or psychotropic drugs (for example antidepressants or anti-anxiety medications)?

- Yes
- No

1. Do you use cannabis?

- Yes
- No

1. How long have you been using CBD?

- 0-3 months
- 3-6 months
- 6-12 months
- 1-2 years
- 2-5 years
- more than 5 years

1. At what time of the day do you use CBD?

- In the morning
- In the evening
- In the morning and the evening
- Multiple times per day (>2)
- When needed

1. How do you usually take CBD? (Choose all that apply)

- Sublingually
- Capsules or Pills
- Topical on the skin
- Vaping
- Edibles
- Smoking
- Sprayed in the mouth
- Suppository
- Other (please specify)

1. How much CBD do you use per day?

- 0-24 mg
- 25-49 mg
- 50-99 mg
- 100-149 mg
- 150-199 mg
- 200 <
- I don't know

1. What was the reason for using CBD? (Choose all that apply)

- For general health and wellbeing
- Stress
- Chronic Pain
- Anxiety
- Headaches/migraine
- Depression
- To increase focus
- To improve sleep
- To improve mood
- Menstrual Pain
- Skin conditions
- Fatigue
- Neurodegenerative disease
- Addiciton (other substance)
- Curiousity
- Other (please specify)

1. Do you think CBD products have effectively helped your ailments?

- Yes
- No

1. Which other benefits and effects do you feel from CBD? (Choose all that apply)

- I have a better mood
- I have a better memory
- I focus better
- I feel more calm
- I feel euphoric/high
- My muscles relax
- Increased energy
- I have no positive benefits from CBD
- Other (please specify)

1. Are you currently using CBD?

- Yes
- No

1. Have you noticed any side effects after using CBD?

- Yes
- No

1. What side effects have you noticed after using CBD?

(please specify)
